# Computational Intelligence and Neuroscience in Neurorobotics

**DOI:** 10.1155/2019/6506802

**Published:** 2019-07-01

**Authors:** Somyot Kaitwanidvilai, Uma Seeboonruang, Hisayuki Aoyama, Khemraj Emrith

**Affiliations:** ^1^King Mongkut's Institute of Technology Ladkrabang (KMITL), Bangkok, Thailand; ^2^The University of Electro-Communications, Tokyo, Japan; ^3^University of West of England, Bristol, UK

Computational intelligence and neuroscience play an important role in robotics applications to improve the robot's capacities not only in increasing productivity in manufacturing but also in intelligence development so that robots can substitute human's thinking and planning capacity. Recently, neuroscience has been applied to robots to generate intelligence models with biological nervous systems. The intersection between robotics and neuroscience highlights many promising approaches and applications, for example, the neurorobots, brain-inspired algorithms and devices in robotics, and neural network-based navigation. AI leads to one of the most significant paradigm shifts and improvements in the robotics field. The principal aim of this special issue is to publish quality papers of new developments and trends, novel techniques, and innovative methodologies on the theories and applications of computational intelligence and neuroscience in robotics. Potential topics include but are not limited to the followings: neural networks for biological and biomedical robots, neuroinspired robot navigation, decision support systems in neurorobotics, intelligent fault detection and identification in neurorobotics, clustering and data analysis in neurorobotics, swarm robotics based on neural networks, and neurofuzzy control design for cooperative robots. The following paragraphs summarize the main contents of the best novelty research papers published in this special issue.

The paper “Coevolution of the Asymmetric Morphology and the Behaviour of Simple Predator Agents in Predator-Prey Pursuit Problem” by M. Georgiev, I. Tanev, and K. Shimohara concentrates on the one of the previous challenges. It introduced a new standpoint to the well-studied predator-prey pursuit problem using an implementation of straightforward predator agents. Genetic algorithm was implemented that results in a successful capture of the prey by the team of predator agents. The results were considered towards the engineering of asymmetric small-scale for delivery of medicine, locating and destroying cancer cells, microscopic imaging, etc.

The paper “Discrimination of Motion Direction in a Robot Using a Phenomenological Model of Synaptic Plasticity” by N. Berberian, M. Ross, and S. Chartier examines the possibility of implementation of a bioinspired model of synaptic plasticity in a robotic agent to allow the discrimination of motion direction of real-world stimuli. The research started with a well-established model of short-term synaptic plasticity (STP), and then the development of a microcircuit motif of spiking neurons capable of exhibiting preferential and nonreferential responses to changes in the direction of an orientation stimulus in motion was introduced. Overall, the model presented the STP function in describing the direction selectivity and applied these in an actual robot to validate the response characteristics in experimental direction selectivity.

The paper “Spatial Concept Learning: A Spiking Neural Network Implementation in Virtual and Physical Robots” by A. Cyr and F. Thériault proposes an artificial spiking neural network (SNN). The SNN sustained the cognitive abstract process of spatial concept learning and embedded in virtual and real robots. The results showed that the robots can learn the relationship of horizontal/vertical and left/right visual stimuli. Tests with novel patterns and locations were effectively completed after the acquisition learning phase. The results also presented that the SNN can change its behavior in real time when the rewarding rule changes.

The paper “Control of a Humanoid NAO Robot by an Adaptive Bioinspired Cerebellar Module in 3D Motion Tasks” by A. Antonietti et al. focuses on a bioinspired adaptive model by a spiking neural network made of thousands of artificial neurons to control a humanoid NAO robot in real time. The model moved forward and encoded as spikes. The generated spiking activity of its output neurons was decoded in order to yield the suitable correction on the motor actuators. With different time scales, three bidirectional long-term plasticity rules have been embedded for different connections. The neurorobot successfully learned how to compensate for the external perturbation generating an appropriate correction during the perturbed upper limb reaching protocol. Hence, the spiking cerebellar model was able to duplicate in the robotic platform how biological systems deal with external sources of error.

The paper “Neurofuzzy c-Means Network-Based SCARA Robot for Head Gimbal Assembly (HGA) Circuit Inspection” by S. Kaitwanidvilai and R. Praserttaweelap describes a decision and control of SCARA robot in HGA (head gimbal assembly) inspection. The method applied a general image processing technique, blob analysis, in conjunction with neurofuzzy c-means (NFC) clustering with the branch and bound (BNB) technique in order to find the best structure in all possible candidates to increase the performance of the entire system. The results from two clustering techniques were investigated to show the effectiveness of the proposed algorithm. Training results from the 30x microscope inspection with 300 samples showed that the best accuracy for clustering was 99.67% from the NFC clustering and for testing results showing 92.21% accuracy for the conventional Kohonen network. This system has been implemented successfully in the HDD production line at Seagate Technology (Thailand) Co. Ltd.

The paper “Multilayer Hybrid Deep-Learning Method for Waste Classification and Recycling” by Y. Chu et al. studied a multilayer hybrid deep-learning system (MHS) to automatically sort waste disposed by individuals in the urban municipal area. This system deployed a high-resolution camera capturing waste image and sensors to detect another useful feature information. The MHS used a CNN-based algorithm to remove image features and a multilayer perceptrons (MLP) method to consolidate image features and other feature information to classify wastes as recyclable or the others. The results presented the overall classification accuracy higher than 90% under two different testing scenarios. This significantly outperformed a reference CNN-based method relying on image-only inputs.

In summary, these six papers have showed the actively practical research topics in Computational Intelligence and Neuroscience in Neurorobotics. We thank all authors for submitting their papers to this special issue and recognize all reviewers for providing their expertise review and excellent comments. We hope that all papers in this special issue would contribute to the research ideas and methodology development in the related fields.

